# The diagnostic role of complete MICM-P in metastatic carcinoma of bone marrow (MCBM) presented with atypical symptoms: A 7-year retrospective study of 45 cases in a single center

**DOI:** 10.1097/MD.0000000000031731

**Published:** 2022-11-11

**Authors:** Chao Wang, Zhiqiong Wang, Xiwen Tong, Yi Li, Xian Liu, Lifang Huang

**Affiliations:** a Department of Hematology, Tongji Hospital, Tongji Medical College, Huazhong University of Science and Technology, Wuhan, Hubei, China; b Hepatic Surgery Center, Institute of Hepato-Pancreato-Biliary Surgery, Department of Surgery, Tongji Hospital, Tongji Medical College, Huazhong University of Science and Technology, Wuhan, Hubei, China.

**Keywords:** biopsy, bone marrow, diagnosis, immunohistochemistry (IHC), metastatic carcinoma of bone marrow (MCBM)

## Abstract

Metastatic carcinoma of bone marrow (MCBM) tends to present with atypical symptoms and can be easily misdiagnosed or miss diagnosed. This study was conducted to investigate the clinical-pathological and hematological characteristics of MCBM patients in order to develop strategies for early detection, staging, treatment selection and prognosis predicting. We retrospectively analyzed 45 patients with MCBM diagnosed by bone marrow biopsy in our hospital during the past 7 years. The clinical symptoms, hemogram and myelogram features, Hematoxylin and eosin staining and immunohistochemistry staining of bone marrow biopsies, location of primary carcinoma and corresponding treatment of the 45 MCBM patients were analyzed in this study. In total, 35 (77.9%) of all patients presented pains including bone pain (73.3%) as the main manifestation, and 37 (82.2%) patients had anemia. Metastatic cancer cells were found in only 22 patients (48.9%) upon bone marrow smear examination, but in all 45 patients by bone marrow biopsy. The bone marrow of 18 (40.0%) patients was dry extraction. Distribution of metastatic carcinoma was diffuse in 20 (44.4%) patients and multi-focal in 25 (55.6%) patients, complicated with myelofibrosis in 34 (75.6%) patients. For bone marrow biopsy immunohistochemistry, 97.8% of the patients were CD45-negative, while 75.6% of the patients were Cytokeratin-positive. There were 30 patients (66.7%) identified with primary malignancies. The overall survival (OS) of 1 year for MCBM patients was 6.7%. There was a trend that patients with cancer of known primary obtained better prognosis according to the survival curve, but the finding was not statistically significant with Log-rank *P* = .160. Complete MICM-P plays a significant role in early diagnosis of MCBM. Bone marrow biopsy combined with immunohistochemistry is an underappreciated method for the diagnosis of MCBM, which should be taken as part of regular tests as well as bone marrow smear. Understanding the clinical-pathological and hematological characteristics of MCBM and conducting bone marrow biopsy in time are of great significance for early detection and treatment selection.

**Key Messages:**

**1.**What is already known on this topic?

Metastatic carcinoma of bone marrow (MCBM) could cause the destruction of bone marrow structure and the disorder of hematopoiesis function, and tend to present with atypical symptoms and can be easily misdiagnosed or miss diagnosed.

**2.**What this study adds?

We retrospectively analyzed 45 patients with MCBM diagnosed by bone marrow biopsy for 7 years in a tertiary hospital and evaluated the diagnostic role of complete MICM-P in metastatic carcinoma of bone marrow (MCBM) without atypical symptoms.

**3.**How this study might affect research, practice or policy?

Bone marrow biopsy combined with immunohistochemistry is an underappreciated method for the diagnosis of MCBM, which should be taken as part of regular tests as well as bone marrow smear. A flowchart of diagnostic routine for MCBM with atypical symptoms has been come up with in this study.

## 1. Introduction

Metastatic carcinoma of bone marrow (MCBM) is a general term for the metastasis of malignant neoplasms from nonhematopoietic origins to bone marrow, which causes the destruction of bone marrow structure and disorder of hematopoiesis function.^[1]^ Bone marrow is a common site of metastasis, which can be the only site of metastasis in certain cases.^[2–4]^ MCBM is one of the rare manifestations of advanced malignant tumors. Patients with MCBM pretend to show hematological abnormalities, such as anemia and thrombocytopenia,^[5]^ but the primary tumor is hard to locate. These cases can be easily misdiagnosed or the diagnosis missed at the first visit. Bone marrow aspiration and/or bone marrow biopsy play an essential role to detect the primary malignancies in this circumstance.^[6]^ Therefore, it is of great significance to investigate the clinicopathological and hematological characteristics of patients with MCBM for early detection, staging, treatment selection and prognosis predicting.

MICM, representing “M (morphology), I (immunology), C (cytogenetics), M (molecular biology),” has been widely accepted for differentiation diagnosis of leukemia by bone marrow aspiration.^[7]^ However, the significant role of complete MICM-P (P for pathology) by bone marrow biopsy is underestimated in diagnosis of hematological malignancies, especially MCBM.^[8,9]^ This study retrospectively analyzed 45 patients with MCBM diagnosed by bone marrow biopsy from March 2014 to October 2021 in a tertiary hospital to evaluate the significance of complete MICM-P in MCBM patients.

## 2. Materials and methods

### 2.1. Participant enrollment

We retrospectively reviewed 11,338 consecutive patients who underwent bone marrow biopsy in our single center, from March 2014 to October 2021. After exclusion of patients with benign blood disorders (72.39%), malignant blood disorders (26.05%) and myeloproliferative neoplasm (1.16%), a total of 45 patients with confirmed diagnosis of MCBM were enrolled into this analysis (Figure S1, http://links.lww.com/MD/H903). The retrospective study involving human participants were reviewed and approved by Medical Ethics Committee of Tongji Hospital, Tongji Medical College, Huazhong University of Science and Technology. The informed consent was waived by the Ethics Committee of Tongji Hospital as the retrospective study.

### 2.2. Data collection

The 45 patients with MCBM were examined by bone marrow aspiration and bone marrow biopsy. Bone marrow fluid was extracted by routine aspiration of the anterior/posterior superior iliac spine. After Wright’s staining of bone marrow smear, the degree of hyperplasia, the percentage of granulocyte and erythrocyte, megakaryocyte count and abnormal cell mass of the bone marrow were examined by low-power microscope. The morphologic characteristics and percentage of nuclear cells in each stage were observed by oil microscope. Bone marrow biopsy was obtained by trephining bone marrow tissue with a length of more than 0.8 cm, followed by formalin immersion, decalcification, paraffin embedding and semithin sectioning. Hematoxylin and eosin staining and immunohistochemistry (IHC) staining of the bone marrow biopsy confirmed the lesion was not originated from the hematological system. The specific antibodies used for IHC staining in this study were cytokeratin (CK), CD138, CD45, BCL2, CD34, CD117, CD68, MPO, CD15, CD3, CD20, and Ki-67. The degree of hyperplasia, the distribution and morphology of abnormal tumor cells, the proportion of hematopoietic cells, bone destruction, and the abnormal expression of markers were observed by low-power microscope. All the data were reviewed in detail by 2 experienced pathologists independently. The clinical symptoms, hemogram and myelogram features, HE staining and IHC staining of bone marrow biopsies, location of primary carcinoma and corresponding treatment of the 45 MCBM patients were analyzed in this study.

### 2.3. Statistical analysis

Continuous variables were expressed as the median and range. Categorical variables were described as numbers (%). Survival curves were depicted using the Kaplan–Meier method and compared using the log-rank test. Statistical analyses were performed by IBM SPSS Statistics version 22.0.

## 3. Results

### 3.1. Basic characteristics of 45 MCBM patients

There were 27 males and 18 females enrolled into this retrospective study with the male to female ratio of 1.5:1. The cohort included 10 patients in 21 to 40 years old, 23 patients in 41 to 60 years old and 12 patients older than 60 years old. The median age was 53 years with the range of 25 to 80 years.

The main symptoms included pain (77.9%), anemia (82.2%), hemorrhage (33.3%), lymphadenopathy (11.1%) and fever (8.9%). Persistent and progressive pain was the dominant complaint among all these atypical systems with bone pain (73.3%) as the most common pain followed by abdominal pain (4.4%) and chest pain (2.2%). 37 patients presented progressive anemia with mild, moderate and severe anemia accounting for 17.8%, 51.1%, and 13.3% of all the patients. 33.3% of the patients suffered from hemorrhage, including 8 patients for subcutaneous hemorrhage, 3 patients for gastrointestinal hemorrhage, and 4 patients for other kinds of hemorrhage. Enlarged lymph nodes were observed in 5 patients presenting in the groin, neck, armpit or supraclavicular fossa. There were 4 patients presenting fever of unknown origin or suboptimal response to antibiotic therapy.

The white blood cell count ranged from 1.45 to 65.95 × 10^9^/L in the 45 MCBM patients. Most leukocytes were classified as neutrophils, some of which had toxic granules in the cytoplasm. The platelet count in 14 patients was within normal range (100–300 × 10^9^/L), but increased in 2 patients. There were 29 patients presenting thrombocytopenia (less than 100 × 10^9^/L) within 18 patients less than 50 × 10^9^/L. Peripheral blood smear showed 48.9% of the patients had immature granulocytes and 28.9% had nucleated red blood cell (Table 1).

**Table 1:** Basic characteristics of 45 MCBM patients.

Item, n (%)	Patients (n = 45)
**Age (yr), median (range**)	53 (25–80)
21–40	10 (22.2%)
41–60	23 (51.1%)
>60	12 (26.7%)
**Gender**	
Male	27 (60.0%)
Female	18 (40.0%)
**Symptom**	
Pain	35 (77.9%)
Bone pain	33 (73.3%)
Abdominal pain	2 (4.4%)
Chest pain	1 (2.2%)
Anemia	37 (82.2%)
Mild	8 (17.8%)
Moderate	23 (51.1%)
Severe	6 (13.3%)
Hemorrhage	15 (33.3%)
Subcutaneous	8 (17.8%)
Gastrointestinal	3 (6.7%)
Other	4 (8.9%)
Lymphadenopathy	5 (11.1%)
Fever	4 (8.9%)
**Complete blood count**	
WBC (×10^9^/L), median (range)	6.55 (1.45–65.95)
<4.0	11 (24.4%)
4.0–10.0	33 (73.4%)
≥10.0	1 (2.2%)
PLT (×10^9^/L), median (range)	69 (3–340)
<50	18 (40.0%)
50–99	11 (24.4%)
100–299	14 (31.2%)
≥300	2 (4.4%)
**Peripheral blood smear**	
Immature granulocyte presenting	
Yes	22 (48.9%)
No	23 (51.1%)
Nucleated red blood cell presenting	
Yes	13 (28.9%)
No	32 (71.1%)

Anemia is classified as severe (<60 g/L), moderate (60–89 g/L), and mild (90–109 g/L) by the level of hemoglobin.

MCBM = metastatic carcinoma of bone marrow, PLT = platelet, WBC = white blood cell.

### 3.2. Analysis of bone marrow smear

Among the 45 patients, the proportions of patients presenting severe hypoplasia, hypoplasia, hyperplasia and marked hyperplasia were 35.5%, 22.4%, 31.1%, and 8.9%, respectively (Table S1, http://links.lww.com/MD/H904). The degree of hyperplasia was determined based on WHO standard.^[10]^ Dry tap on bone marrow aspiration was found in 18 patients (40.0%).

Granulocyte percentage decreased in 14 patients (31.1%) and erythrocyte percentage decreased in 21 patients (46.7%). There were 4 patients showing hypochromic microcytic, which were further confirmed to be iron deficiency anemia by iron staining (data not shown). Megakaryocytes was severely reduced or even unable to detect in 28 patients (62.2%), but the other 17 patients (37.8%) showed active or significantly active hyperplasia. 2 patients (4.44%) were found to present significant megakaryocyte mature hindrance (data not shown).

**Figure 1.**
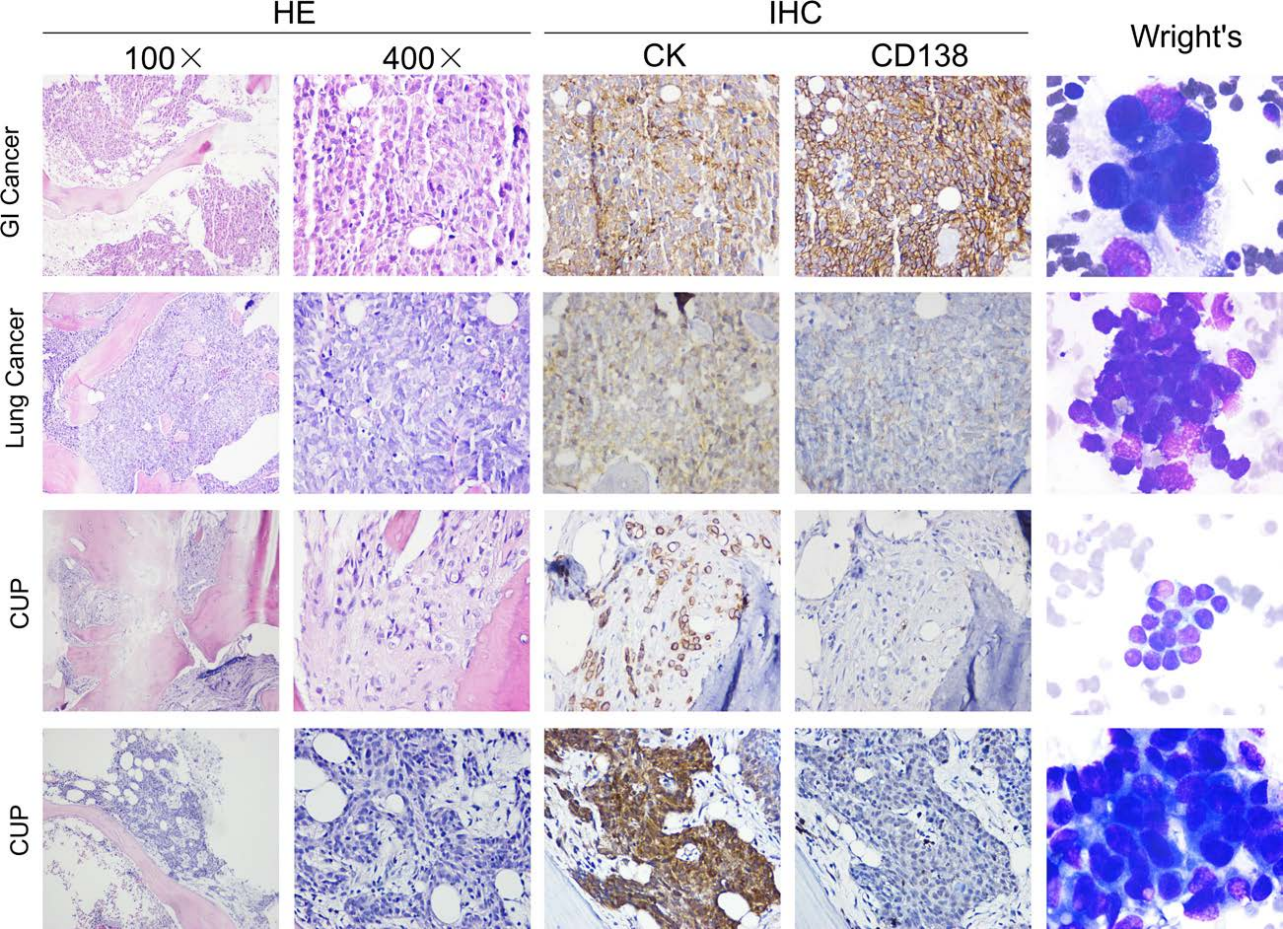
HE, IHC and Wright’s staining for bone marrow biopsy and smear in representative cases of gastrointestinal cancer, lung cancer and cancer of unknown primary. HE = hematoxylin and eosin, IHC = immunohistochemistry.

Tumor cells (unable to be identified with the specific origins) were observed in 22 patients (48.9%) by the bone marrow cytological smears. In most of these patients (42.2%), abnormal cells clustered at the tail and edge of the smear with irregular arrangement. These large sized cells were round or quasi-round in shape, and the membrane edge was torn, pulled and incomplete, with blue cytoplasm and large nucleus. Two or more nucleoli could be observed in certain cases. There was also a small number of patients (6.7%) presenting with scattered unidentified cells, which can be easily ignored due to the mixture with normal bone marrow cells (Fig. 1).

### 3.3. Analysis of bone marrow biopsy

The 45 patients with MCBM enrolled into our study were all confirmed by bone marrow biopsy, while 23 of them failed to be diagnosed by bone marrow aspiration. Thirty-four patients were accompanied by myelofibrosis, including 13 patients with diffused distribution and 21 patients with multi-focal distribution. The IHC staining showed negative expression of MPO, CD15, CD3, and CD20 in all patients, and negative expression of CD45 in 97.8% of the patients. The positive rates of CK, CD138, BCL2, and Ki-67 were 75.6%, 33.3%, 31.1%, and 11.1%, respectively (Table 2).

**Table 2:** The result of bone marrow biopsy.

Item, n (%)	Patients (n = 45)
**Distribution of metastatic carcinoma**	
Diffuse	20 (44.4%)
With myelofibrosis	13 (28.9%)
Without myelofibrosis	7 (15.5%)
Multi-focal	25 (55.6%)
With myelofibrosis	21 (46.7%)
Without myelofibrosis	4 (8.9%)
**CK**	
Positive	34 (75.6%)
Negative	11 (24.4%)
**CD138**	
Positive	15 (33.3%)
Negative	29 (64.5%)
NA	1 (2.2%)
**CD45**	
Positive	1 (2.3%)
Negative	44 (97.8%)
**BCL2**	
Positive	14 (31.1%)
Negative	24 (53.3%)
NA	7 (15.6%)
**Ki-67**	
Positive	5 (11.1%)
Negative	35 (77.8%)
NA	5 (11.1%)
**CD34**	
Positive	1 (2.2%)
Negative	44 (97.8%)
**CD117**	
Positive	5 (11.1%)
Negative	40 (88.9%)
**CD68**	
Positive	4 (8.9%)
Negative	41 (91.1%)
**MPO**	
Positive	0 (0.0%)
Negative	45 (100.0%)
**CD15**	
Positive	0 (0.0%)
Negative	45 (100.0%)
**CD3**	
Positive	0 (0.0%)
Negative	45 (100.0%)
**CD20**	
Positive	0 (0.0%)
Negative	45 (100.0%)

NA = not available.

### 3.4. Treatment and prognosis for the 45 patients with MCBM

Hematological abnormalities, along with chief complaints of bone pain, anemia and/or hemorrhage, tended to raise the suspicion of multiple myeloma or hematologic malignancy. After consulting with hematologists, MCBM was diagnosed by bone marrow aspiration and biopsy. The primary lesions were identified in 30 patients (66.7%), including 9 patients with gastrointestinal cancer, 6 patients with lung cancer, 5 patients with breast cancer, 2 patients each with rhabdomyosarcoma, nasopharyngeal carcinoma, prostate cancer and neuroblastoma, and 1 patient each with vascular endothelial tumor and ovary cancer after further targeted examination (Table 3).

**Table 3:** Primary cancer and chemotherapy.

Item, n (%)	Total	Chemotherapy	
		Yes	No
**MCBM with known primary**	**30 (66.7%**)	**12 (26.7%**)	**18 (40%**)
Gastrointestinal cancer	9 (20.0%)		
Lung cancer	6 (13.3%)		
Breast cancer	5 (11.1%)		
Rhabdomyosarcoma	2 (4.4%)		
Nasopharyngeal carcinoma	2 (4.4%)		
Prostate cancer	2 (4.4%)		
Neuroblastoma	2 (4.4%)		
Vascular endothelial tumor	1 (2.2%)		
Ovary cancer	1 (2.2%)		
**MCBM with unknown primary**	**15 (33.3%**)	**6 (13.3%**)	**9 (20%**)

MCBM = metastatic carcinoma of bone marrow.

**Figure 2.**
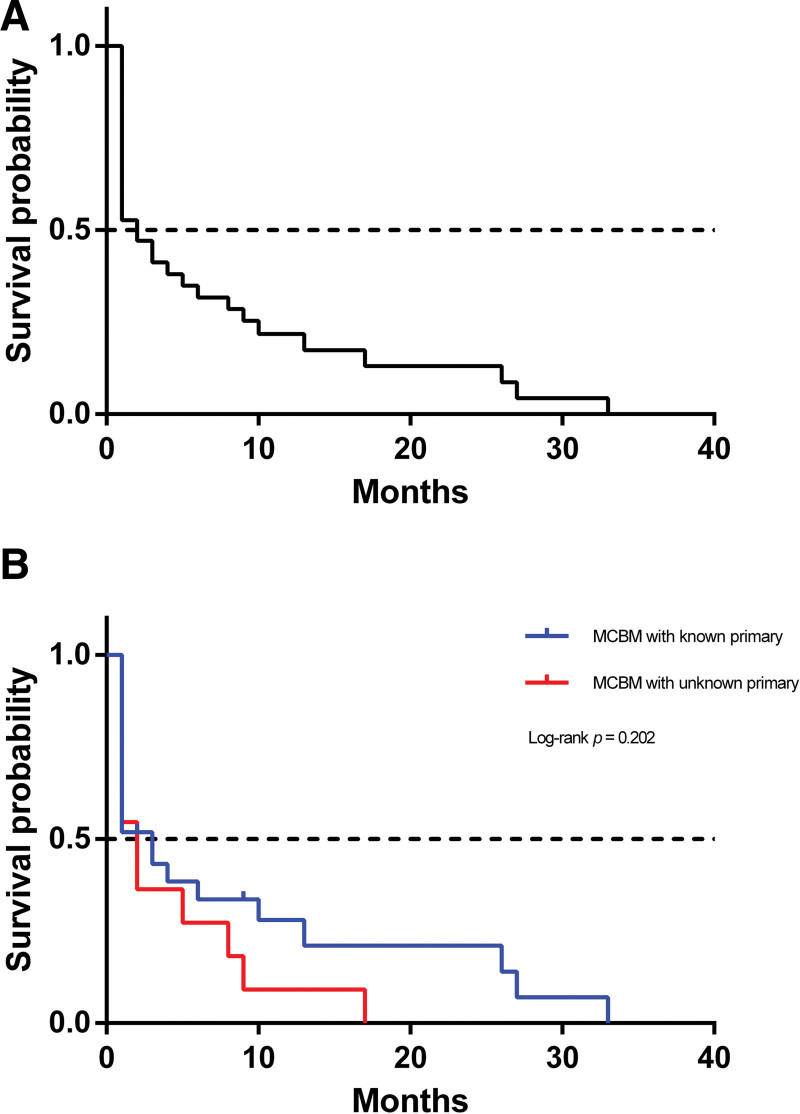
Kaplan–Meier survival curves of overall survival in patients with MCBM. (A) OS for all MCBM patients; (B) OS comparing MCBM patients with/without known primary cancer. MCBM = metastatic carcinoma of bone marrow, OS = overall survival.

Among the 30 MCBM patients with known primary cancer, 12 patients underwent chemotherapy, while only 6 patients received chemotherapy out of 15 patients with cancer of unknown primary (CUP) (Table 3). The overall survival (OS) curve for MCBM patients was presented in Figure 2. To explore the potential influence of known or unknown primary cancer on OS, we analyzed the 2 groups with Kaplan–Meier method. Though there was a trend that patients with cancer of known primary obtained better prognosis according to the survival curve, the finding was not statistically significant with Log-rank *P* = .160.

## 4. Discussion and conclusions

Bone marrow is an important targeted organ of malignant tumor metastasis, following liver and lungs.^[11]^ In some cases, metastasis to the bone marrow occurs earlier than other organs, which could be the only site of metastasis. Bone marrow metastasis has been recognized as a poor prognostic factor for cancer patients. Therefore, early detection and early treatment are of vital importance to improve prognosis of MCBM patients. Bone marrow metastasis often starts insidiously with various atypical symptoms, including bone pain, anemia, hemorrhage and others. Li et al found that 80% of cancer patients suffered from varying degrees of bone pain, which was the primary reason for MCBM patients to seek medical treatment.^[12]^ Patients with severe anemia and osteolytic damage share similar clinical manifestations of multiple myeloma. Anemia is one of the most common manifestations in patients with solid tumor bone marrow metastasis.^[13,14]^ In cancer patients there were a number of coagulation abnormalities, providing the background for an increased tendency of thrombosis and hemorrhage.^[15]^ Sallah S et al reported a 7% incidence of disseminated intravascular coagulation occurrence in 1117 patients with solid tumors.^[16]^ In our study, the main symptoms of MCBM are anemia (82.2%), bone pain (73.3%), hemorrhage (33.3%) and fever (8.9%). Due to these atypical symptoms, most of the MCBM patients came to orthopedic or respiratory departments at their first medical visit, or did not take the manifestation seriously.

MICM has been widely applied in hematology fields, but complete MICM-P is still under-appreciated, especially for clinicians who do not specialize in hematology. The complete MICM-P is useful for diagnosing, staging and predicting prognosis of various solid tumors by complementing each element.^[14,17,18]^ In our study, metastatic tumor cells were found in 45 patients by bone marrow biopsy, but only 22 patients positive by bone marrow aspiration, indicating bone marrow pathology was more sensitive than cytology in detecting MCBM. Bone marrow aspiration provides information about the numerical and cytological features of bone marrow cells, whereas bone marrow trephine biopsy achieves excellent appreciation of spatial relationships between cells and of overall bone marrow structure.^[19]^ For cases with unexplained hypoplasia detected by bone marrow aspiration cytology, hematologists must carry out a bone marrow biopsy to avoid missed diagnosis or misdiagnosis.

IHC staining has been widely applied for further identifying. The canonical IHC antibodies include CD34, CD117, MPO, CD68, CD15, CD3, CD20, CD138, CK, CD45, BCL2, and Ki-67. CD34 was not only a marker of bone marrow primitive cells in the early stage but also overexpressed in the vascular endothelium, indicating the abundant blood vessels in MCBM.^[20]^ CD138 was widely expressed in multiple myeloma, but the specificity was limited.^[21]^ In this study, 15 of the metastatic cancer patients (33.3%) were CD138-positive, suggesting the deficient role in differentiating metastatic carcinoma from multiple myeloma. BCL2 and Ki-67 were proliferative markers expressed in metastatic cancer, though the positive rate was not significant.^[22,23]^ CD45 was the common antigen of leukocytes. 97.8% of the patients were CD45-negative, indicating the metastasis malignancies mainly originated from solid tumor instead of blood system.^[22]^ One patient was both CD45 and CK positive, which was rare. The main symptom of this patient was back pain accompanied by skin bleeding. At the first visit, the patient’s blood routine was significantly abnormal, and she was highly suspicious of leukemia originally and was diagnosed as MCBM by MICM-P, but she failed to find the primary lesion, and died 1 month later. Kota Ishizawa et al reported higher proportions of CD45/CD326 doubly-positive cells in lung cancer tissue were significantly associated with poor prognosis.^[24]^ Meanwhile, 75.6% of the metastatic cancers in this study were CK-positive. CK, a common tumor marker expressed in epithelial cells, is the best option for bone marrow metastasis screening via IHC staining.^[25]^ Positive result of CK can confirm epithelial metastasis, while negative result cannot exclude non-epithelial metastasis. In our study, the primary tumors of patients were rhabdomyosarcoma (2 cases), neuroblastoma (2 cases) and vascular endothelial tumor (1 case), which were CK negative. Other specific antibodies for IHC staining can be introduced to identify primary tumors hard to be diagnosed by other kinds of examination, including prostate specific antigen for prostate carcinoma,^[26]^ gross cystic disease fluid protein-15 for primary lung neoplasms,^[27]^ estrogen receptor for breast carcinomas,^[28]^ thyroid transcription factor-1 (TTF-1) for medullary thyroid carcinomas.^[29]^ Chandra Krishnan et al reported that the IHC characterization of metastatic carcinoma in bone marrow had good correlation with the established staining pattern of the primary tumors.^[30]^

The distribution mode of metastatic carcinoma in the bone marrow was diffuse and multifocal growth, making it difficult to confirm the morphology of tumor cells. Tissue extrusion was not rare in the process of bone marrow biopsy. Bone marrow metastasis, in particular, hires proliferation of reticular and collagen fibers. Among the 45 cases, 33 were complicated with bone marrow fibrosis in diagnosis. Bone marrow fibrosis was seen in a variety of malignancies (e.g., multiple myeloma and myelodysplastic syndrome) and non‐malignancies (e.g., chronic autoimmune diseases, infections, and exposure to radiation or toxins).^[31,32]^ It was likely that both malignant and non‐malignant diseases activate fibrosis‐driving cells by common downstream mechanisms.^[33]^ Dry tap refers failure to obtain bone marrow on attempted marrow aspiration.^[34,35]^ It was common in metastatic carcinoma, chronic myelogenous leukemia, idiopathic myelofibrosis and hairy cell leukemia.^[36]^ In this study, 18 cases presented dry tap or limited extraction of bone marrow fluid. No cancer cells were found in these extractions. Extensive marrow fibrosis and hypercellularity have been proposed as mechanisms to account for the inability to withdraw marrow by aspiration.^[36,37]^ In 45 patients who underwent bone marrow biopsy simultaneously, 31 patients were accompanied with bone marrow fibrosis. Pathological changes were likely to happen due to the marrow dry tap and bone marrow dilution during the aspiration operation. Practitioners should not cease the examination and exclude the diagnosis easily. The positive detection rate of bone marrow aspiration in patients with bone pain can be improved by puncturing smear at the site of pain or sampling at multiple sites.

For the small parts of patients with clear primary focus, good general condition, but without obvious hematological abnormality, proper treatment for the primary tumor could be achieved in time. However, missed diagnosis or misdiagnosis of primary tumor, which takes the large proportion in MCBM, usually cause a prolonged delay before the effective treatment and unfavored prognosis further. Therefore, even if the prognosis of bone marrow metastases is poor, the earlier detection and treatment can also prolong the survival time and quality of life of patients. In recent years, most researches have focused on the treatment of carcinoma of unknown primary.^[38–40]^ Simplified gemcitabine, oxaliplatin, leucovorin and 5-fluorouracil regimen appears to be feasible with promising activity for CUP in whom immunohistostaining was suggestive of either upper gastrointestinal or pancreato-biliary cancers.^[40]^ In our study of 45 MCBM cases, the primary tumor focus was found in 30 patients, of which 12 received chemotherapy and 18 received supportive treatment. 15 patients failed to find the primary focus, of which 6 cases received chemotherapy, and the other 9 cases received supportive treatment. The OS of the MCBM patients with known primary was not better than that with unknown primary (*P* = .22). Most of the patients suffered from multiple site metastasis, with progressive failure of the bone marrow and the whole body due to their advanced age. Generally, the patients were suffering from anemia, thrombocytopenia and drug resistance in unsatisfying economic situation. Palliative care including supportive and symptomatic treatments was given to relieve symptoms and improve life quality of these patients.^[41]^ The prognosis was dismal, with the short survival of several days to 3 months since the diagnosis of MCBM. This study was carried in a single center. Further studies from multiple centers with larger population are required to validated the results.

**Figure 3.**
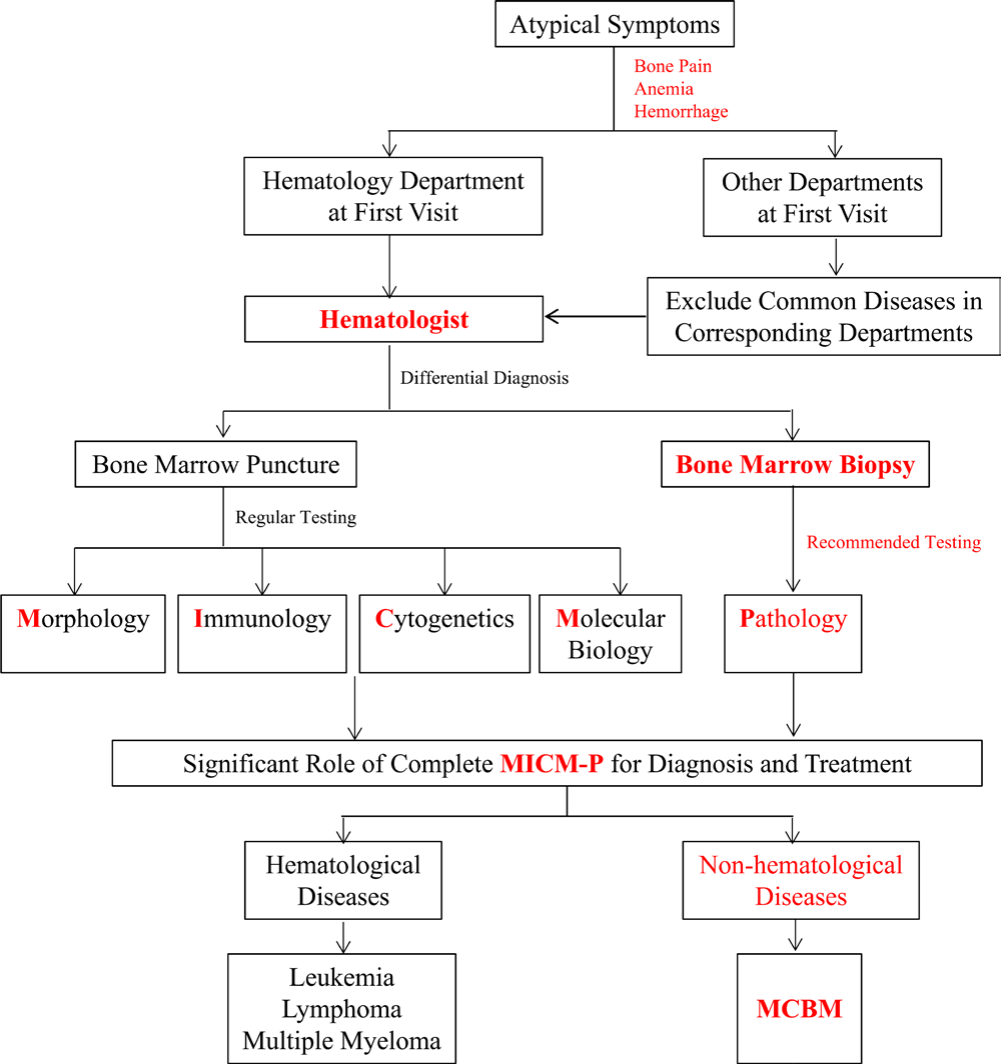
Flowchart of MCBM diagnosis routine. MCBM = metastatic carcinoma of bone marrow.

Bone marrow aspiration and biopsy are essential in diagnosis for certain malignancies or non-malignancies; however, they were rarely considered in non-hematology departments due to the insufficient awareness of specialists to exclude blood diseases or MCBM. We suggest following procedures for patients with atypical symptoms of bone pain, anemia and hemorrhage to improve the diagnosis accuracy of MCBM:

1. For the patients visiting the non-hematology departments presenting anemia, bone pain and/or hemorrhage, common diseases in corresponding departments should be excluded before referral to hematologists.
2. Bone marrow puncture and biopsy are combined to provide concrete evidence of morphology, immunology, cytogenetics, molecular biology and pathology for differential diagnosis.
3. Following treatments are applied according to hematological and non-hematological diseases determined by the complete MICM-P (Fig. 3).

In conclusion, understand the clinical-pathological and hematological characteristics is crucial for early and accurate detection of MCBM in patients with atypical symptoms. Complete MICM-P improves the diagnosis of MCBM comparing with traditional MICM-P. MCBM should be included in the differential diagnosis for all patients presenting atypical symptoms, particularly anemia, bone pain and hemorrhage.

## Acknowledgments

All authors have contributed to, read and approved the final manuscript. We thanked Dr Bin-hao Zhang from Hepatic Surgery Center, Tongji Hospital for his valuable advices in this study.

## Author contributions

**Conceptualization:** Zhiqiong Wang, Chao Wang, Lifang Huang.

**Data curation:** Zhiqiong Wang, Xiwen Tong, Yi Li, Xian Liu.

**Formal analysis:** Zhiqiong Wang, Xiwen Tong, Xian Liu.

**Funding acquisition:** Chao Wang.

**Methodology:** Zhiqiong Wang, Chao Wang, Xiwen Tong, Yi Li.

**Writing – original draft:** Zhiqiong Wang, Chao Wang, Lifang Huang.

**Writing – review & editing:** Lifang Huang, Chao Wang, Zhiqiong Wang.

## Supplementary Material


