# Supplementary figures and images for: The diagnostic role of complete MICM-P in metastatic carcinoma of bone marrow (MCBM) presented with atypical symptoms: A 7-year retrospective study of 45 cases in a single center
Source: Medicine (Baltimore). 2022 Nov 11;101(45):e31731. doi: 10.1097/MD.0000000000031731 (PMC9666179; doi:10.1097/MD.0000000000031731)

**Supplementary Figure 1.** Flowchart of the patient selection.

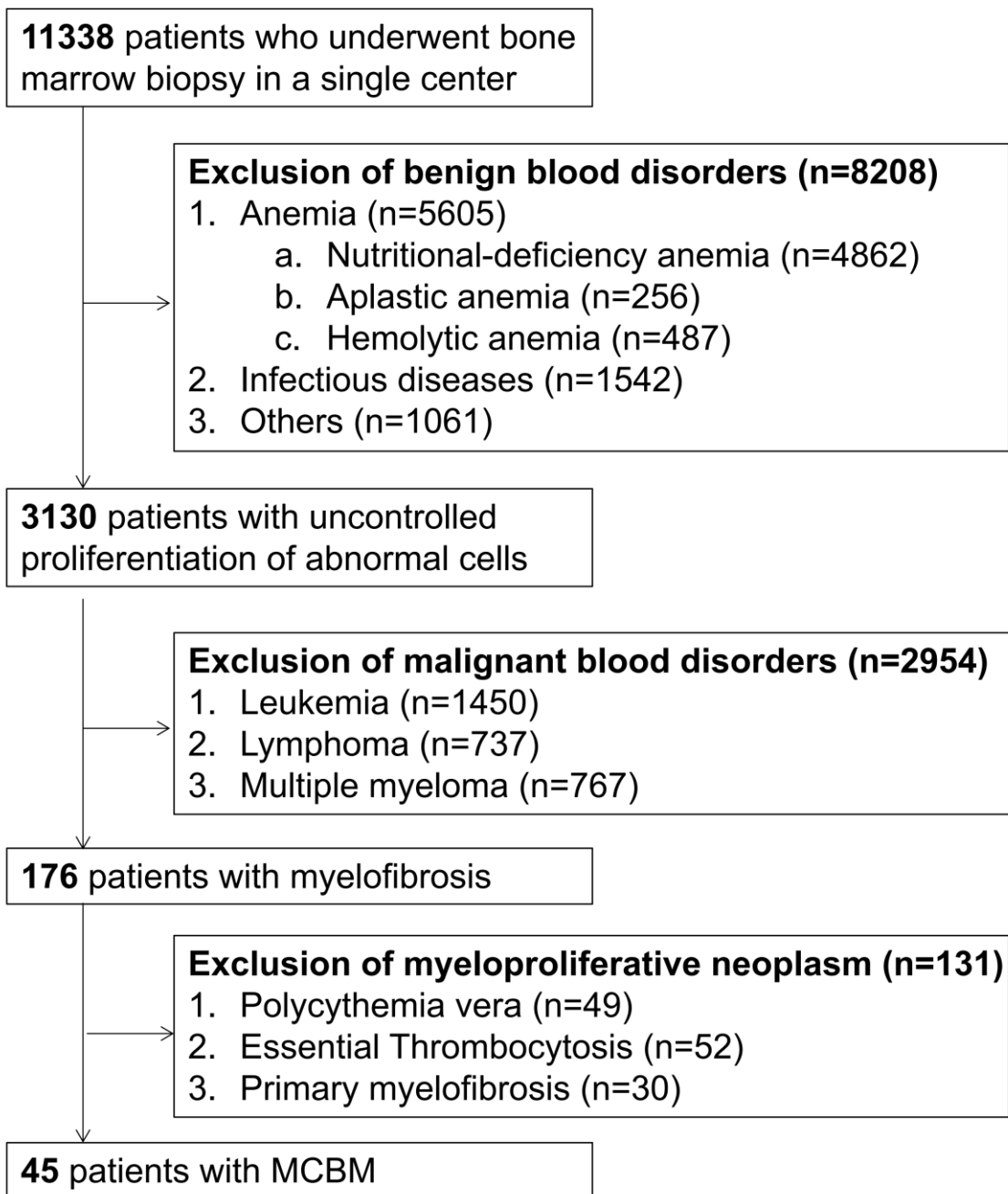

Supplement: Supplementary file 1 [file medi-101-e31731-s001.pdf]
