# Supplementary material for: The diagnostic role of complete MICM-P in metastatic carcinoma of bone marrow (MCBM) presented with atypical symptoms: A 7-year retrospective study of 45 cases in a single center
Source: Medicine (Baltimore). 2022 Nov 11;101(45):e31731. doi: 10.1097/MD.0000000000031731 (PMC9666179; doi:10.1097/MD.0000000000031731)
Supplement: Supplementary file 2 [file medi-101-e31731-s002.pdf]

Supplementary Table 1. The result of bone marrow smear

Item, n (%)	Patients (n=45)
**Degree of hyperplasia**	
Severe hypoplasia	16 (35.5%)
Hypoplasia	11 (22.4%)
Hyperplasia	14 (31.1%)
Marked hyperplasia	4 (8.9%)
**Dry tap**	
Yes	18 (40.0%)
No	27 (60.0%)
**Granulocyte percentage**	
＜45%	14 (31.1%)
⩾45%	31 (68.9%)
**Erythrocyte percentage**	
＜15%	21 (46.7%)
⩾15%	24 (53.3%)
**Megakaryocyte count**	
＜20	28 (62.2%)
⩾20	17 (37.8%)
**Tumor cells presenting**	
Yes	22 (48.9%)
Clustered	19 (42.2%)
Scattered	3 (6.7%)
No	23 (51.1%)
